# Political consciousness, agency and mental health. Students in the Covid 19-Pandemic: Yasemin Niephaus

**DOI:** 10.1093/eurpub/ckac131.547

**Published:** 2022-10-25

**Authors:** Y Niephaus, T Nink

**Affiliations:** Department of Social Sciences, University Siegen, Siegen, Germany

## Abstract

**Introduction:**

The influence of social crises in their objective quality as well as their subjective processing on health well-being has been researched many times. The loss of agency that can be observed in the course of social crises is considered to be a negative mediating factor between both variables. The Corona pandemic was also associated with a loss of options and routines for action for most of the population.

**Methods:**

We conducted two quantitative surveys on mental health in the pandemic in spring 2020 and autumn 2021. While the first international comparative survey was conducted under the auspices of the University of Antwerp, the second was carried out at five German universities.

**Results:**

The quantitative data show the negative impact of the pandemic on students’ mental health - CES-D 8 (0 to 24) (x¯2020=9.22, x¯2021=9.38). They also show a negative correlation between whether students’ concerns are sufficiently considered by policy makers in the pandemic and depressive symptomatology (r=-0.146, p < 0.01). That is, the less satisfied students are with political measures, the lower the depressive symptomatology.

**Conlusion:**

Against the background of the socio-psychological inequality study ‘Die Arbeitslosen von Marienthal', which is considered a classic, this connection is far less paradoxical than might initially be assumed if one interprets political awareness as a correlate of a subjectively perceived power to act, which has a positive effect on mental health. Based on this thesis, we have opted for a mixed-methods design and are conducting qualitative interviews on subjective crisis processing, the results of which we will be able to present at the conference.

**Key messages:**

• Subjectively perceived agency reduces the health-reducing effect of social crises.

• Students are a relevant group to learn from for capacity building.

